# A weekly time-weighted method of outdoor and indoor individual exposure to particulate air pollution

**DOI:** 10.1016/j.mex.2019.10.013

**Published:** 2019-10-18

**Authors:** Xin Liu, Moran Dong, Jiaqi Wang, Dengzhou Chen, Jianpeng Xiao, Weilin Zeng, Xing Li, Jianxiong Hu, Guanhao He, Wenjun Ma, Tao Liu

**Affiliations:** a Guangdong Provincial Institute of Public Health, Guangdong Provincial Center for Disease Control and Prevention, Guangzhou, 511430, China; b General Practice Center, Nanhai Hospital, Southern Medical University, Foshan, 528200, China

**Keywords:** Weekly time-weighted air pollution exposure assessment method, Air pollution, Exposure assessment, Land use regression model, Preterm birth

## Abstract

The aim of this study was to estimate the weekly time-weighted (outdoor and indoor activity patterns) individual exposure to particulate air pollutants (PM_10_, PM_2.5_ and PM_1_) of pregnant women. A total of 4928 pregnancy women were recruited during their early pregnancy, and 4278 (86.8%) were successfully followed-up at childbirth. Each individual weekly average PM_10_ and PM_2.5_ concentrations at the residential and workplace addresses from three months before pregnancy to childbirth was estimated using a spatiotemporal land use regression (ST-LUR) model, and the weekly PM_1_ concentration was estimated employing a generalized additive model (GAM) which utilized weekly PM_2.5_ and meteorological factors as independent predictors. Then, the time-weighted individual exposure to particulate air pollutants during workdays and non-workdays during the period from three months before pregnancy to childbirth was estimated based on the estimated weekly air pollutant concentrations and each participant’s indoor and outdoor activity model, respectively. Data analysis was carried out by R software (version 3.5.1) and packages “SpatioTemporal”, “mgcv” and “splines” were mainly used. This method takes a full consideration of indoor and outdoor activity patterns in the individual exposure to particulate air pollutants.

• A ST-LUR model was used to estimate the individual weekly average PM_10_ and PM_2.5_ concentrations at their residential and workplace addresses.
• A GAM was applied to estimate the weekly average PM_1_ concentration at individual residential and workplace addresses.
• Individual weekly exposure to particulate air pollutants during workdays and non-workdays was assessed based on the estimated particulate air pollutant concentrations and their indoor and outdoor activity model.

## Graphical abstract

**Figure:**
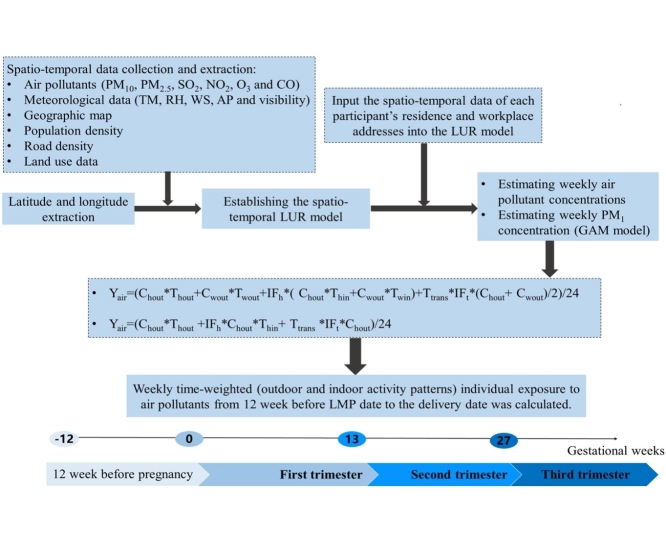

**Specification Table**

**Table:** 

Subject Area:	Environmental Science
More specific subject area:	Guangdong province, South China
Method name:	Weekly time-weighted air pollution exposure assessment method
Name and reference of original method:	Chen R, Zhou B, Kan H, Zhao B. 2013. Associations of particulate air pollution and daily mortality in 16 Chinese cities: an improved effect estimate after accounting for the indoor exposure to particles of outdoor origin. Environ Pollut 182:278-82. doi: 10.1016/j.envpol.2013.07.024 Zhou X, Cai J, Zhao Y, Chen R, Wang C, Zhao A, et al. 2018. Estimation of residential fine particulate matter infiltration in Shanghai, China. Environ Pollut 233:494-500. doi: 10.1016/j.envpol.2017.10.054
Resource availability:	Data

## Method details

In this study, we conducted a birth cohort study on Prenatal Environments and Offspring Health (PEOH Cohort) in Guangzhou, China since 2016. We aimed to estimate the weekly time-weighted (outdoor and indoor activity patterns) individual exposure to particulate air pollutants (PM_10_, PM_2.5_ and PM_1_) during pregnancy, and further prospectively estimate the effects of prenatal exposures to particulate air pollutants with different sizes on the risk of PTB. We selected the Guangzhou Panyu Central Hospital as the study setting which is the largest hospital in Panyu district of Guangzhou. All pregnant women were initially recruited from the outpatient department of obstetrics if they met the following criteria: (1) their gestational weeks were ranged from 1 to 13; (2) 18–50 years old; (3) did not have the following diseases: hyperthyroidism, heart disease, chronic kidney disease, tuberculosis, psychiatric disease and other serious diseases. A total of 4928 pregnant women were recruited in the baseline investigation, and 4278 (86.8%) were successfully followed-up during the hospitalization for childbirth. We have obtained each participants’ family addresses and workplace addresses, transport activity patterns, indoor and outdoor activity patterns. The PEOH study was approved by the Ethics Committee of Guangdong Provincial Center for Disease Control and Prevention. Every recruited participant was given a detailed introduction and explanation of this study, and signed the informed consent.

The data of daily ambient air pollutant (PM_10_ and PM_2.5_) of 102 monitoring stations across Guangdong province were collected from the National Urban Air Quality Real-time Publishing Platform (http://106.37.208.233:20035/) (January 1 st 2014 to December 31 st 2017), and meteorological data (daily mean temperature ([TM)], relative humidity ([RH)], mean wind speed ([WS)], atmospheric pressure ([AP]), and visibility) of all 86 monitoring stations from Guangdong Meteorological Service. Geographic information system (GIS) covariates (geographic map, road density, and land use data) were obtained from the Data Center for Resources and Environmental Sciences (http://www.resdc.cn), and population density data in 2015 from GeoData Institute in University of Southampton (www.worldpop.org.uk). Then, a spatiotemporal land-use-regression (ST-LUR) model was established to estimate weekly ambient concentrations of PM_10_ and PM_2.5_ at each individual residential and workplace addresses from three months before pregnancy to childbirth using visibility, population density, road length and land use data as predictors [1]. The weekly PM_1_ concentrations at individual residential and workplace addresses were estimated employing a generalized additive model (GAM) which included the weekly PM_2.5_ concentration and meteorological factors (TM, RH, AP and WS) as predictors [2,3]. We have described the detailed process of PM_10_ and PM_2.5_ exposure assessment in our previous study [4].

Based on the above estimated particulate air pollutants concentrations, we used the Eq. (1) to estimate the time-weighted individual exposure to air pollution during workdays, and employed the Eq. (2) to assess the exposure during non-workdays based on each participant’s indoor and outdoor activity time [5]. For each individual, the weekly exposure (from the first day of the corresponding gestational week to the following 6 days) from 12 weeks before the last menstrual period (LMP) date to the week at delivery was calculated.

(1) Y_air_=(C_hout_*T_hout_+C_wout_*T_wout_+IF_h_*(C_hout_*T_hin_+C_wout_*T_win_)+T_trans_*IF_t_*(C_hout_+ C_wout_)/2)/24

(2) Y_air_=(C_hout_*T_hout_ +IF_h_*C_hout_*T_hin_+ T_trans_ *IF_t_*C_hout_)/24

**Fig. 1:**
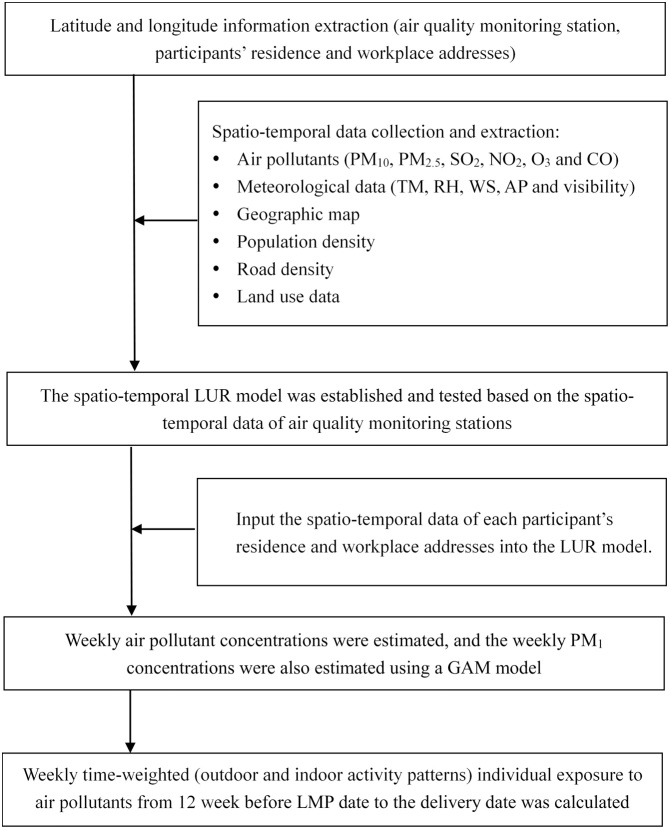
The process of individual exposure assessment to air pollutants.

In which, *Y_air_* denotes the time-weighted air pollution concentrations of each individual; *C_hout_* denotes the average concentrations of air pollutant at each participant’s residence address which was estimated above; *C_wout_* denotes the average concentration of ambient air pollutants at each participant’s work place which was also estimated above; *T_hout_* denotes the daily average hours spent outdoors around their residence; *T_wout_* denotes the daily average hours spent outdoors around their work place; *T_hin_* denotes the daily average hours spent indoors at their home; *T_win_* denotes the daily average hours spent indoors in their work place; *T_trans_* denotes the daily average hours spent on transportation; *IF_h_* denotes the infiltration factor (0.83) of particulate air pollutants infiltrating into indoor environments from outdoors [6]; *IF_t_* denotes the infiltration factor of particulate air pollutants, which varies for different types of transportation. *(C_hout_+C_wout_)/2* denotes the average concentration of particulate air pollutants at residence and workplace. Here, we employed it to represent the particulate air pollutant concentration for transportation. The number of 24 denotes 24 h a day, it means a day time-weighted air pollution concentrations of each individual (Table 1). The performance of the modeling is shown in Fig. 1. We have used this method to estimate the exposures in our study [7].

**Table 1:** Infiltration factor of PM_2.5_ for different types of transportation.

Type of transportation	Infiltration factor of PM_2.5_
Walking^a^	1
Bicycling^a^	1
Electric bicycles^a^	1
Motor vehicle (bus or car) [8]	0.66
Metro [8]	0.62

a Participants were directly exposed to the ambient PM_2.5_.

All statistical analyses were performed using R software (version 3.5.1), and packages “SpatioTemporal”, “mgcv” and “splines” were used [9].

## Declaration of Competing Interest

The authors declare no conflict of actual or potential competing financial interests.
